# Comment on Matsumoto et al. Remimazolam’s Effects on Postoperative Nausea and Vomiting Are Similar to Those of Propofol after Laparoscopic Gynecological Surgery: A Randomized Controlled Trial. *J. Clin. Med.* **2023**, *12*, 5402

**DOI:** 10.3390/jcm13040923

**Published:** 2024-02-06

**Authors:** Kai Su, Fu-Shan Xue, Yi Cheng

**Affiliations:** Department of Anesthesiology, Beijing Friendship Hospital, Capital Medical University, No. 95 Yong-An Road, Xi-Cheng District, Beijing 100050, China

**Keywords:** laparoscopic gynecological surgery, postoperative nausea and vomiting, remimazolam, propofol, general anesthesia

The recent article published in this journal by Matsumoto et al. [1], comparing the postoperative antiemetic efficacy of remimazolam and propofol as general anesthetics in patients who underwent laparoscopic gynecological surgery, was of great interest to us. They showed that remimazolam was as effective as propofol in preventing postoperative nausea and vomiting (PONV). Given that PONV remain common and distressing postoperative complications with delayed recovery and the increased use of healthcare resources [2], their findings have potential implications. Besides the limitations described by the authors in their discussion section, however, there are several issues in this study that are not well addressed.

First, based on the difference in the incidence of PONV between sevoflurane- and propofol-based anesthesia regimens reported in previous work, the authors selected a reduction in PONV incidence of 34% as the effect size to evaluate the sample size of this study. Available evidence indicates that inhalational anesthesia is a known risk factor of PONV, and the incidence of PONV is significantly lower when using propofol- and remimazolam-based anesthesia regimens than when using inhalational anesthesia [3,4,5]. Given that the main aim of this study is to compare the influences of two intravenous anesthetics with significant antiemetic effect on the occurrence of PONV, we are concerned with the use of data from the studies comparing the incidence of PONV between inhalational anesthesia and propofol-based anesthesia as the effect size would have significantly underestimated the sample size of this study and resulted in a type 2 statistical error possibility for their findings.

Second, this is a randomized controlled trial and no significant difference might have been expected, but the authors did not provide whether the two groups were comparable with respect to preoperative fasting time. It has been shown that prolonged preoperative fasting duration is associated with an increased risk of PONV [6]. Furthermore, the occurrence of intraoperative hypotension was not provided, though it was defined in their methods section. The recent work in female patients undergoing cervical conization demonstrates that remimazolam-based general anesthesia is associated with a decreased incidence of intraoperative hypotension compared with propofol-based general anesthesia [7]. Most importantly, intraoperative hypotension has been associated with an increased risk of PONV [8]. Thus, we believe that addressing these unknown factors would improve the transparency of this research design and the interpretation of the results.

Third, for postoperative analgesia, regional blocks were performed and both acetaminophen and flurbiprofen were administered at the end of surgery. However, it was unclear why two non-opioid analgesics were not regularly administered postoperatively and only opioids were used as rescue analgesics, though PONV are the most common opioid-related-adverse effects. Just like the authors described in their article, the objects of this study were at a high risk of PONV as they were female patients undergoing laparoscopic gynecological surgery, their mean age was 50 years or less, and about 20% of them had a history of motion sickness or PONV. For the high-risk patients for PONV, we would like to remind the authors and readers that an opioid-sparing multimodal analgesia strategy has been recommend as a component of the multimodal prophylaxis for PONV [2,9]. Other than regional blocks, an opioid-sparing multimodal analgesia strategy actually contains a combination of acetaminophen and nonsteroidal anti-inflammatory drugs or cyclooxygenase-specific inhibitors and requires that these non-opioid analgesics are administered postoperatively on schedule, unless contraindicated. Opioids should be used sparingly as rescue analgesics only when non-opioid basic analgesics are ineffective. The use of a non-standardized opioid-sparing multimodal analgesia strategy may not significantly change the primary outcomes of this study, but we argue that clarification of this issue is useful for improving the design quality of randomized clinical trials comparing the postoperative antiemetic efficacy of different interventions.
